# A Novel Insertion Variant of *CRYGD* Is Associated with Congenital Nuclear Cataract in a Chinese Family

**DOI:** 10.1371/journal.pone.0131471

**Published:** 2015-07-06

**Authors:** Xiaotong Zhuang, Lianqing Wang, Zixun Song, Wei Xiao

**Affiliations:** 1 Department of Ophthalmology, Shengjing Hospital, China Medical University, Shenyang, China; 2 Department of Ophthalmology, The Fourth People’s Hospital of Shenyang, Shenyang, China; 3 Department of Medical Genetics, Institute of Basic Medical Sciences, Chinese Academy of Medical Sciences & Peking Union Medical College, Beijing, P. R. China; 4 Central Laboratory, Central Hospital of Zibo, Zibo, China; Tsinghua University, CHINA

## Abstract

**Objective:**

To investigate a novel insertion variant of *CRYGD* identified in a Chinese family with nuclear congenital cataract.

**Methods:**

A Chinese family with congenital nuclear cataract was recruited for the mutational screening of candidate genes by direct sequencing. Recombinant N-terminal Myc tagged wildtype or mutant CRYGD was expressed in HEK293T cells. The expression pattern, protein solubility and subcellular distribution were analyzed by western blotting and immunofluorescence.

**Principal Findings:**

A novel insertion variant, c.451_452insGACT, in *CRYGD* was identified in the patients. It causes a frameshift and a premature termination of the polypeptide to become Y151*. A significantly reduced solubility was observed for this mutant. Unlike wildtype CRYGD, which existed mainly in the cytoplasm, Y151* was mis-located in the nucleus.

**Conclusions:**

We have identified a novel mutation, c.451_452insGACT, in *CRYGD*, which is associated with nuclear cataract. This is the first insertion mutation of *CRYGD* found to cause autosomal dominant congenital cataract. The mutant protein, with loss of solubility and localization to the nucleus, is hypothesized to be the major cause of cataract in these patients.

## Introduction

Congenital cataracts are a major cause of visual impairment and blindness in childhood [1]. The estimated prevalence is 1–6 per10,000 live births, approximately one-third of which are believed to be inherited [2, 3]. Inherited cataracts are clinically highly heterogeneous, commonly exhibiting considerable inter- and intrafamilial phenotypic variation [4]. In non-consanguineous populations, inherited cataracts most frequently show an autosomal dominant pattern of inheritance, while autosomal recessive and X-linked patterns have also been reported [2, 5]. To date, more than 20 independent genes on different chromosomes have been shown to be associated with isolated congenital cataracts [6]. Among the congenital cataract families with causative mutations discovered so far, about half have mutations in crystallins, with the remainder divided among the genes for gap junction proteins, transcription factors, membrane transporter proteins, and cytoskeletal proteins [7].

Nuclear cataract, which was first described by Brown in 1924, is one of the most familiar types of severe congenital cataract [8, 9]. The visual acuity is remarkably reduced in patients with this kind of cataract because the opacity is located at the center of the lens [10]. Currently, autosomal dominant congenital nuclear cataracts have been linked to 11 specific causative genes [7], and most of the mutations are in the crystallin family of genes [9, 11–15].

In the present study, a four-generation Chinese family with congenital nuclear cataract was recruited. We identified a novel four-base insertion, namely c.451_452insGACT, in exon 3 of *CRYGD* after sequencing the candidate crystallin genes. This change led to a truncated protein Tyr151*. The mutant CRYGD exhibited reduced solubility, and localized incorrectly to the cell nucleus. To our knowledge, this is the first insertion mutation of *CRYGD* found to be disease-causing in congenital cataract.

## Methods

### Study samples

**Fig 1:**
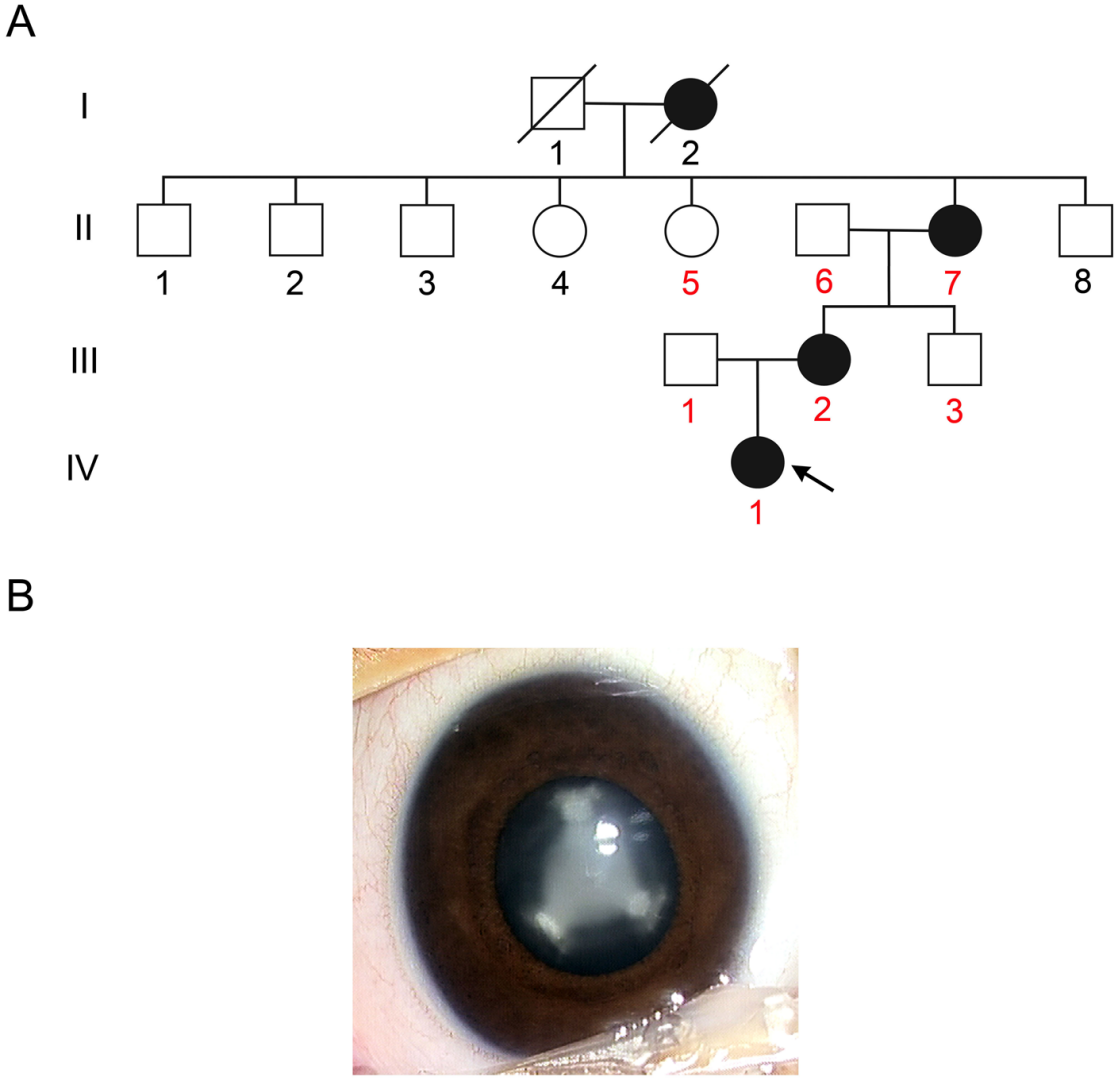
The family with congenital nuclear cataract. (A) Pedigree of the Chinese family with autosomal dominant nuclear cataract. Squares and circles indicate males and females, respectively. Black symbols represent individuals with a cataract phenotype and open symbols represent unaffected individuals. The proband is marked by an arrow. The numbers of the participants in this study are indicated by red color. (B) Lens picture from the proband showed the opacities located in the lens nucleus and that the sutures were also involved (right eye).

We collected blood samples from seven members (three affected and four unaffected) of a four-generation Han Chinese family with autosomal dominant nuclear cataract (Fig 1A) after obtaining informed consent and approval of the China Medical University Review Board. One hundred and three unrelated individuals were also recruited as study controls. Peripheral blood samples were collected for DNA analysis.

### Mutation screening and sequence analysis

Genomic DNA was extracted from 500 μL of peripheral blood using a TIANamp Blood DNA Midi Kit (Tiangen, Beijing, China). After genomic polymerase chain reaction (PCR) performed, we sequenced the coding exons and their flanking intronic sequences of six crystallin family genes CRYAA, CRYBA1, CRYBB1, CRYBB2, CRYGC and CRYGD, for pathogenic mutations in the proband. The primers and reaction conditions used in PCR have been described in earlier reports [16–21]. To more clearly show the mutation c.451_452insGACT in *CRYGD*, the obtained fragment was cloned into pMD-18T vector (TaKaRa, Dalian, China) and sequenced. To confirm the mutation, we performed genomic polymerase chain reaction (PCR) using primers CRYGD forward (5’-TACGAGCTGTCCAACTACCGAG-3’) and reverse (5’-GAGAAATCTATGACTCTCCTCAG-3’) in the family and 103 unrelated individuals. The PCR fragments were separated by 8% polyacrylamide gel electrophoresis. The analysis of amino acid conservation around the mutation site was carried out by CLC DNA Workbench.

### Minigene assay

Genomic DNA fragments containing partial exon 2, intron 2 and partial exon 3 (the sequence before AAUAAA signal) were generated by PCR using primers CRYGD-2Fm (5’-ATCAGGATCCGACTATGCCGACCACCAG-3’) and CRYGD-3Rm (5’-TGAGAATTCAGTTTCCAAATTAAGAAACAAC-3’). The PCR fragments were cloned into the vector pcDNA3.1 (+) (Invitrogen, California, USA) at the *Bam*H I and *Eco*R I sites. The constructs with the wildtype or mutant genomic inserts were verified by direct sequencing. The constructs were individually transfected into HEK293T or SRA 01/04 cells. For RT-PCR, total RNA was isolated using the Trizol reagent (Invitrogen) from the transfected cells and reverse transcription was performed using the PrimeScript 1st Strand cDNA Synthesis Kit (TaKaRa). PCR was then carried out with primers CRYGD-2FS (5’-GACTATGCCGACCACCAGCAG-3’) and pcDNA-R (5’-AACTAGAAGGCACAGTCGAGG-3’). The PCR products were sequenced using the primer CRYGD-2FS.

### Bioinformatics analysis

The effect of the mutation c.451_452insGACT on splicing was predicted by Augustus (http://augustus.gobics.de/). The secondary structures of CRYGD were predicted by the GOR4 Secondary Structure Prediction Method. The protein physical and chemical parameters of the CRYGD protein were analyzed by ProtParam (http://web.expasy.org/protparam/). We also used the BEST/COREX server (http://best.bio.jhu.edu/BEST/index.php) to predict the stability of the wildtype and mutant proteins.

### Plasmid constructs

The human *CRYGD* (GeneBank NM_006891.3) cDNA, encoding 174 amino acid residues, was obtained by PCR amplification from a Human Multiple Tissue cDNA Panel (Ovary tissue, BD, New Jersey, USA) and cloned into pCMV-Myc vector (Clontech, New Jersey, USA) at the site of *EcoR* I and *Bgl* II action. The primers used in the *CRYGD* construct were: sense 5’-TCCTGAATTCCCATGGGGAAGATCACCCTC-3Y and antisense 5Y-GGATAGATCTTCAGGAGAAATCTATGACTC-3’. The Y151* mutant was constructed by site-directed mutagenesis with the following primer: sense 5’-CTGCTGATGCCAGGGGACTGACTATAGGCGCTAC-3’; antisense 5’-GTAGCGCCTATAGTCAGTCCCCTGGCATCAGCAG-3’ (the underline indicates the position of the base insertion). All the constructs were confirmed by direct sequencing.

### Cell culture and transfection

Human embryonic kidney (HEK) 293T cells and human lens epithelium lines SRA 01/04 (Cell Resource Center, IBMS, CAMS&PUMC) were routinely grown in Dulbecco’s modified Eagle medium (DMEM) supplemented with 10% FBS at 37°C with 5% CO_2_. For transfection with plasmid DNA, the cells were cultured on six-well plates at 50% confluence, grown overnight, then transfected with the constructs using Lipofectamine 2000 (Invitrogen).

### Protein over-expression analysis

After 36 h of transfection, the cultured HEK293T cells were harvested and lysed in RIPA buffer (1% NP-40, 0.5% deoxycholate, 0.1% SDS) (Beyotime, Shanghai, China) containing protease inhibitor cocktail (Roche, Basel, Switzerland) for 1h at 4°C. Centrifuge the samples at 12,000×g for 10 min at 4°C. The supernatant was collected and denatured in SDS loading buffer (TaKaRa). The precipitants containing insoluble proteins were washed three times with ice-cold PBS, sonicated, and denatured in SDS loading buffer containing 6 mol/L urea. After boiling, the protein samples were separated by 12% SDS-polyarcylamide gel electrophoresis (PAGE) and then transferred to PVDF membranes (Millipore, Massachusetts, USA). The membranes were blocked with TBS buffer (pH 7.4) containing 0.05% Tween-20 and 5% nonfat milk. The blotted proteins were probed with anti-Myc antibody (9E10) (Santa Cruz Biotechnology, Santa Cruz, USA) or anti-β-actin antibody (ZSGB-Bio, Beijing, China), followed by incubation with Goat anti-Mouse IgG (H+L) (peroxidase conjugated) (Pierce, Illinois, USA). The blots were developed subsequently with an enhanced chemiluminescence (ECL) system (Pierce).

### Immunofluorescence

HEK293T cells grown on glass coverslips were fixed with 4% paraformaldehyde (Sigma Aldrich, Shanghai, China), permeabilized with 0.05% Triton-X 100, and blocked with 5% bovine serum albumin (BSA). Anti-Myc antibody (9E10) incubations were carried out in 5% BSA at a dilution of 1:200. After incubation, the cells were washed with PBS three times. The secondary antibody conjugated DyLight 488 (EarthOx, California, USA) (1:800) was added; this had been tested and no cross-reactivity found. The nucleus was stained with 500 ng/ml DAPI (Sigma Aldrich). Following three final washes with PBS, coverslips were mounted in antifade reagent (Beyotime). The samples were examined by fluorescence microscopy equipped with a DP controller system (DP70, Olympus, Japan).

## Results

### Clinical features

We ascertained a four-generation Chinese family with autosomal dominant nuclear congenital cataracts (Fig 1A). All affected individuals showed nuclear cataract, which presented several months after birth. The visual acuity was impaired gradually as the lens opacity increased, and was seriously reduced at 12–13 years old. We failed to get the visual acuity of proband because she is in early childhood (S1 Table). The affected individuals showed an opacity in the central nucleus region of both lenses. The Y-sutures were also involved with prominent opacity (Fig 1B). No systemic disease or other ocular disease was found.

### Mutation confirmation in *CRYGD*

**Fig 2:**
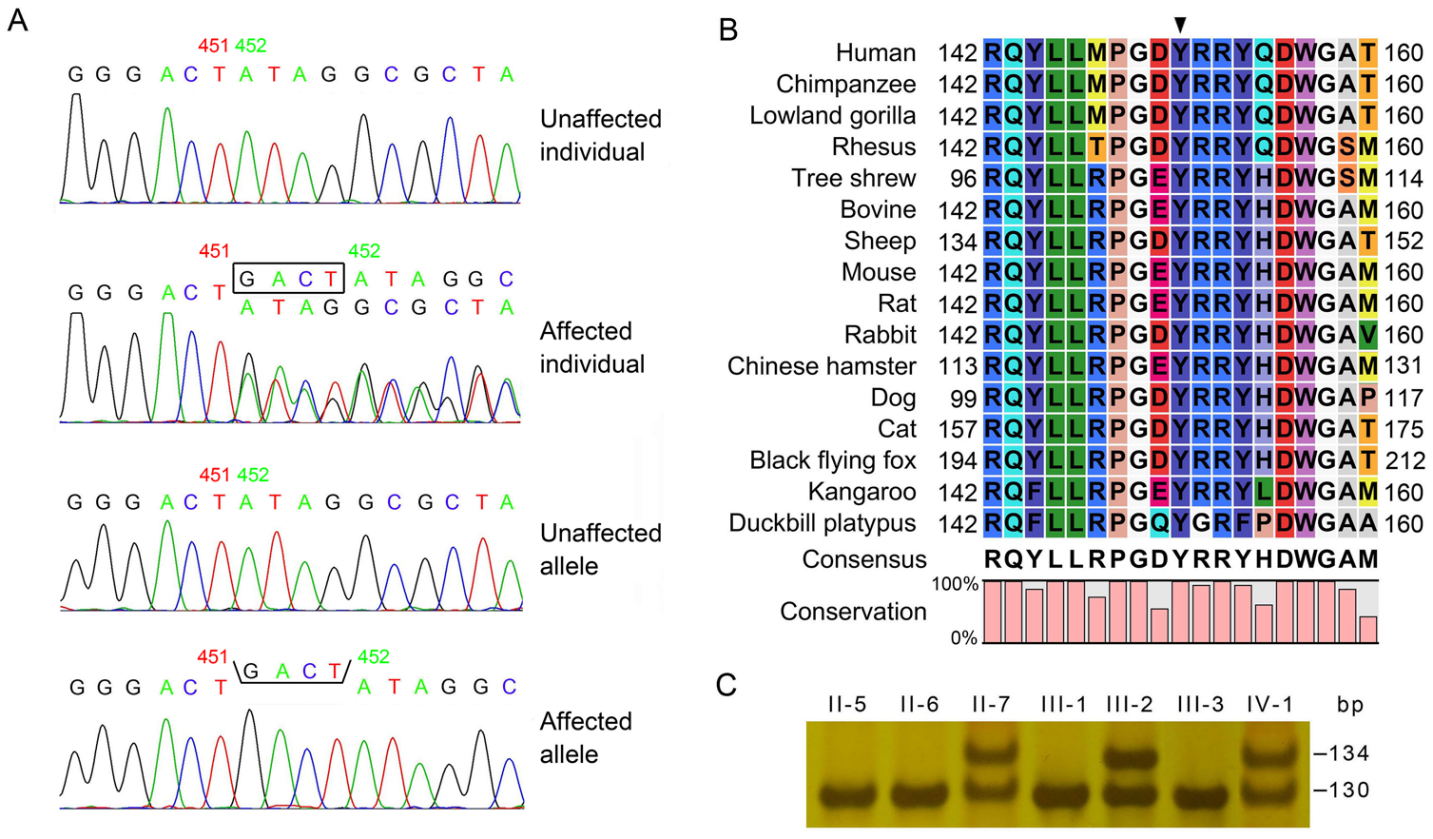
Confirmation of the c.451_452insGACT (p.Tyr151*) insertion mutation of *CRYGD*. (A) Sequence chromatogram showing the heterozygous c.451_452insGACT insertion mutation of *CRYGD* in the proband. The mutation was numbered according to GenBank NM_006891.3. (B) Polyacrylamide gel electrophoresis showing different sizes of the PCR fragments in the pedigree. The 134 bp and 130 bp fragments were amplified from affected and unaffected chromosome, respectively. (C) Protein alignment of mammalian samples showing that the regions around the mutation are highly conserved. Numbers on left and right indicate the position of this fragment. The position of the mutant is marked by a black triangle.

Mutation screenings were performed for all six candidate crystallin genes, and a heterozygous variant, c.451_452insGACT, was identified in exon 3 of *CRYGD* (Fig 2A). The variant led to the substitution of a newly formed stop codon for a phylogenetically conserved tyrosine residue (p.Tyr151*) (Fig 2B), and was only identified in the affected individuals. With the exception of several nonpathogenic SNPs, no other variants were detected. On polyacrylamide gel electrophoresis, the variant was confirmed in all affected individuals but was not detected in unaffected family members or 103 unrelated Chinese controls (Fig 2C).

### The mutation has no effect on splicing

To investigate whether c.451_452insGACT has effect on splicing, it was predicted by Augustus. It showed the splicing was not affected by the mutation (data not shown). To confirm the result, minigene assay was performed. Using primers from exon 2 and pcDNA 3.1, PCR was carried out to analyze the transcription fragments. There were no obvious difference in the length between wildtype and mutant (Figure B in S1 File). Consistent with the results of bioinformatics analysis, splicing was not affected by the mutation c.451_452insGACT, which was confirmed by direct sequencing (Figure C in S1 File).

### Bioinformatics analysis of Y151* on protein level

**Fig 3:**
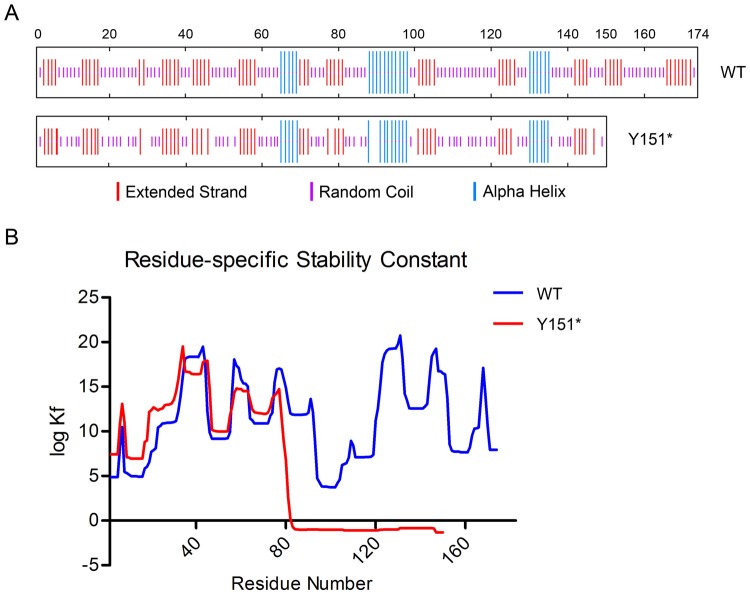
Bioinformatics analysis of the mutant CRYGD Y151*. (A) The predicted secondary structure showing the reduced extended strand and random coil in the mutant. (B) The C-terminal domain of the truncated CRYGD favors the unfolded state. The residue-specific stability constant (K_f_) for each residue of the protein was predicted by the BEST/COREX server.

The mutant protein was truncated by 24 amino acids at the C-terminus when compared with the wildtype, and the fourth Greek key motif (amino acid 129–171) was partially absent. Both the extended strand and the random coil were reduced, resulting in destruction of the secondary structure (Fig 3A). The truncated CRYGD had an acidic isoelectric point (PI), which may be involved in protein aggregation. The mutation rendered CRYGD more unstable and decreased its solubility (Table 1). Moreover, the stability constant (K_f_) at the per residue level showed that the mutant CRYGD favored the unfolded states at the C-terminus (Fig 3B).

**Table 1:** Comparison of CRYGD wildtype and mutant.

Protein characteristics	WT	Y151*
**Number of amino acids**	174	150
**Theoretical PI**	7.00	6.05
**Instablity index**	54.89	56.82
**Aliphatic index**	63.85	63.67
**Grand average of hydropathicity**	-0.804	-0.799

The physical and chemical parameters of the protein were analyzed by ProtParam.

### The mutant CRYGD has decreased solubility

**Fig 4:**
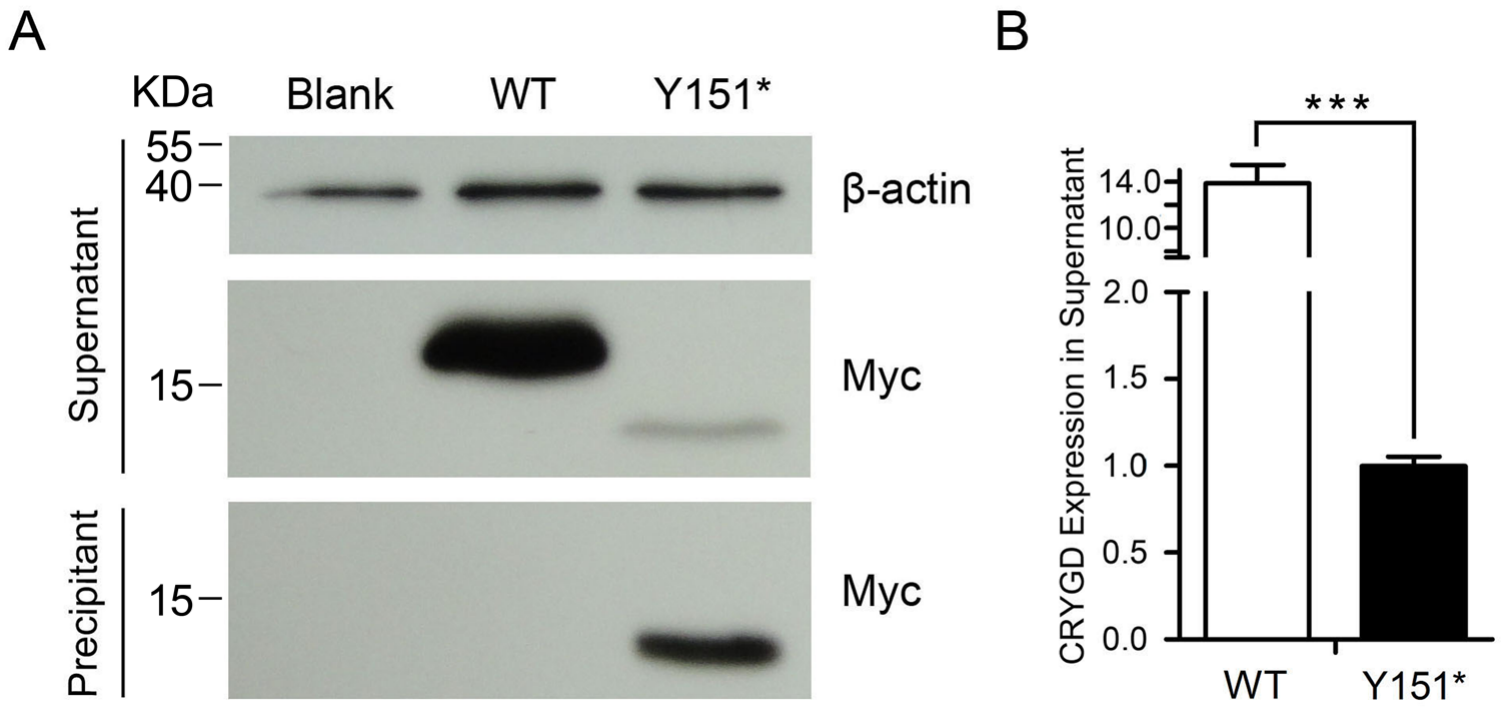
Western blot analysis of CRYGD over-expression in HEK293T cells. (A) The truncated CRYGD showed decreased solubility. In the supernatant, the mutant protein was truncated and was present in much lower amounts than the wildtype. In the precipitant, there was more mutant protein and the wildtype was not detected. (B) Quantification by band densitometry indicated the prominent reduction of mutant CRYGD in the supernatant (p<0.05).

Myc-tagged wildtype and Y151* CRYGD constructs were transiently expressed in HEK293T cells and were detected using anti-Myc antibody (9E10). Western blotting showed that the molecular weight of recombinant mutant CRYGD had decreased as a result of the loss of 24 amino acids (Fig 4A, upper). Moreover, the mutant CRYGD had a greatly reduced level of protein level in the supernatant when compared with wildtype (Fig 4A, upper; Fig 4B). The mutant protein aggregated mainly in the precipitant where the wildtype was not detected (Fig 4A, lower). The results indicated that the mutant protein possessed decreased solubility.

### Mis-localization of mutant CRYGD to the cell nucleus

**Fig 5:**
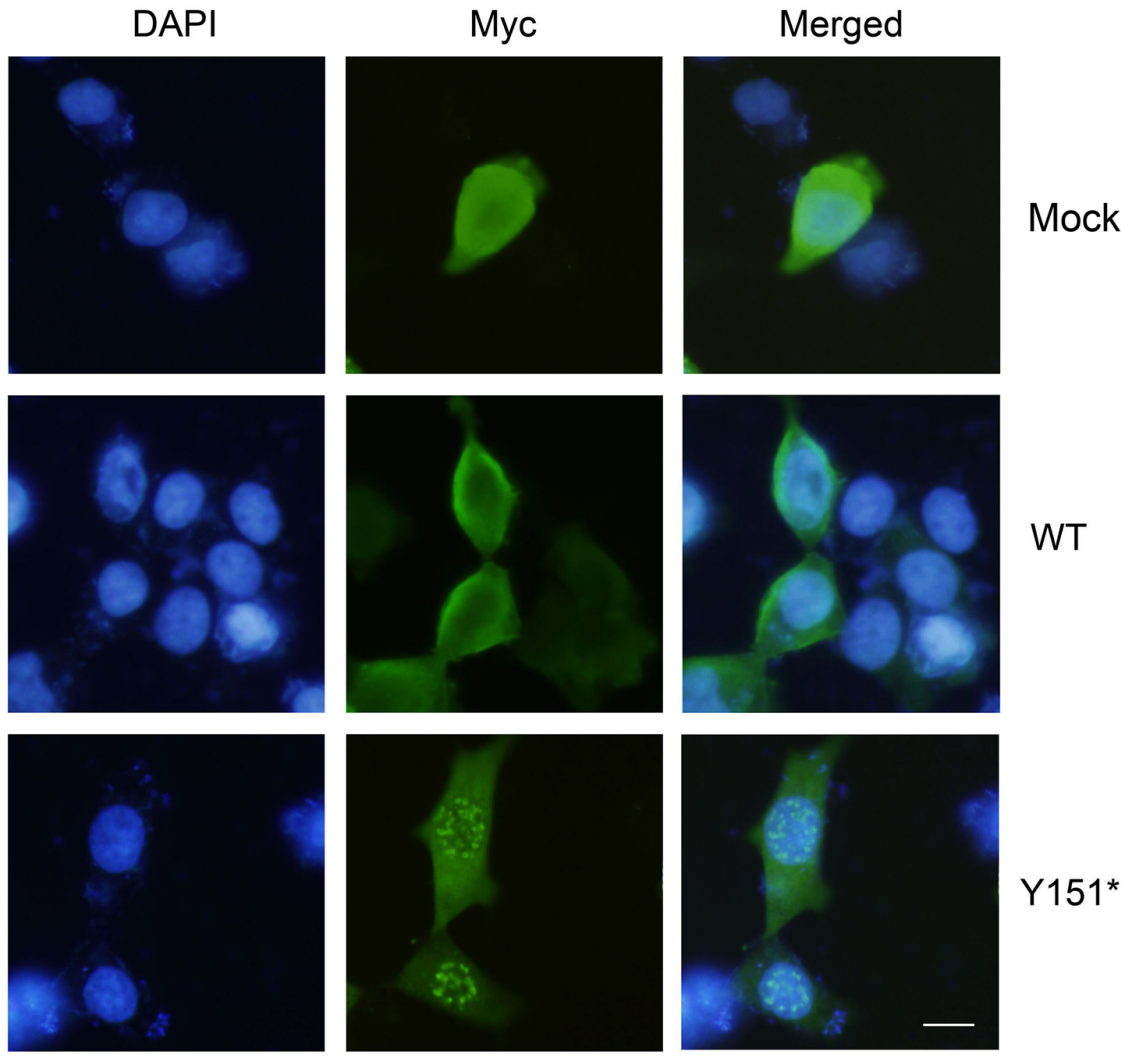
Localization of Myc-tagged wildtype or Y151* CRYGD in HEK293T cells. Immunofluorescence of Myc (green fluorescence) showed the distribution of wildtype CRYGD in both cytomembrane and cytoplasm. However, the mutant CRYGD was mainly localized in the nucleus, in the form of granular deposits. Scale bar: 10 μm.

To confirm the pathogenicity of the mutation Y151*, immunofluorescence assay was performed to detect the subcellular localization of mutant CRYGD. Myc-tagged wildtype CRYGD was localized in both cytoplasmic and membrane regions, whereas Y151* was redistributed in the nucleus as a spot-shaped structure (Fig 5). Minimal staining in the cytoplasm was also observed. The aggregation of the mutant protein in the nucleus showed that it failed to form crystallin and perform its intrinsic function.

## Discussion

In this study, we identified a novel deletion mutation (c.451_452insGACT) in *CRYGD*, which caused a truncated polypeptide (p.Tyr151*). Our expression studies demonstrated that the mutant was highly insoluble and had an abnormal location. The mutant CRYGD may affect lens fiber differentiation thus interfering with lens transparency [22].

Lens transparency is maintained mainly by structural crystallins. Crystallins consist of a core set of three protein families, the α-, β- and γ-crystallins, which are defined by the sizes of the oligomers that they form [23]. Alteration of both the function and the molecular properties of crystallins may cause the breakdown of lens microstructure, which results in changes in the refractive index and increased light scattering [22]. γD-crystallin, encoded by *CRYGD*, is one of the important crystallins. An impairment in γD-crystallin expression may be responsible for cataract [3, 22].

γD-crystallin is composed of two individual domains that each consist of two Greek key motifs (GKM) [3]. Each GKM is composed of four antiparallel β-strands. Up to 2015, a total of 19 mutations in *CRYGD* were reported responsible for cataracts, most of which are missense mutations. However, little functional importance of the mutants was illustrated in the previous reports (S2 Table.). All the mutations distribute in the four GKMs. Intriguingly, the truncated mutations are mainly in the GKM4 of the C-terminus (5/7) (S1 Fig). However, the nonsense mutation c.51T>G (Y17*) and c.168C>G (Y56*) Also, the Y151* mutation is located in the same domain. The insertion c.451_452insGACT in exon 3 of *CRYGD* is predicted to cause a premature stop codon, which leads to the deletion of the C-terminal residues 151–174 (Fig 3A). As shown in the diagram (Fig 3B), the truncated protein destabilizes the C-terminal domain. Moreover, the aggregation state of the mutant γD-crystallin was also affected (Fig 5). Several usually buried residues of the mutant γD-crystallin may be exposed on the surface, which means that the protein displays lower solubility and structural stability [24]. The loss of the C-terminal tail in γD-crystallin leads to substantial intermolecular aggregation, which generates light-scattering particles [25].

It is unclear why the aggregated mutant γD-crystallin was located in the nucleus. There are no amino acid residues related to nuclear localization in the lost C-terminal tail. Asp156, Arg163 and Arg168, the charged residues of wildtype γD-crystallin, are involed in charge interactions [26]. They may play an important role in the processing and subcellular localization of the protein [27]. It will be intriguing to explore how the incorrect localization may have resulted from alteration of the physicochemical properties of the protein.

In summary, we have identified c.451_452insGACT as a novel causative mutation in *CRYGD* responsible for autosomal dominant congenital nuclear cataract. Our results provide a novel insight into the molecular mechanisms involving CRYGD that underlie the pathogenesis of human congenital cataract.

## Supporting Information

S1 Fig The distribution of mutations in *CRYGD* responsible for cataracts. (TIF)

S1 File The effect of the mutation c.451_452insGACT on splicing. The schematic diagram of DNA fragments used in the constructs (**Figure A**). Agarose gel electrophoresis showing no difference in the PCR fragments between wildtype and mutant (**Figure B**). Sequence chromatogram showing the c.451_452insGACT mutation has no effect on splicing (**Figure C**).
(TIF)

S1 Table Clinical information of the family members. (PDF)

S2 Table γD-crystallin mutations, related phenotypes and functional changes. (PDF)

## References

[1] Zhang T, Hua R, Xiao W, Burdon KP, Bhattacharya SS, Craig JE, et al Mutations of the EPHA2 receptor tyrosine kinase gene cause autosomal dominant congenital cataract. Human mutation. 2009;30(5): E603–E611. 10.1002/humu.20995 19306328

[2] Zhai Y, Li J, Zhu Y, Xia Y, Wang W, Yu Y, et al A nonsense mutation of gammaD-crystallin associated with congenital nuclear and posterior polar cataract in a Chinese family. Int J Med Sci. 2014;11(2): 158–163. 10.7150/ijms.7567 24465161 PMC3894400

[3] Nandrot E, Slingsby C, Basak A, Cherif-Chefchaouni M, Benazzouz B, Hajaji Y, et al Gamma-D crystallin gene (CRYGD) mutation causes autosomal dominant congenital cerulean cataracts. J Med Genet. 2003;40(4): 262–267. 12676897 10.1136/jmg.40.4.262 PMC1735438

[4] Santana A, Waiswol M, Arcieri ES, Cabral de Vasconcellos JP, Barbosa de Melo M. Mutation analysis of CRYAA, CRYGC, and CRYGD associated with autosomal dominant congenital cataract in Brazilian families. Mol Vis. 2009;15: 793–800. 19390652 PMC2671581

[5] Scott MH, Hejtmancik JF, Wozencraft LA, Reuter LM, Parks MM, Kaiser-Kupfer MI. Autosomal dominant congenital cataract. Interocular phenotypic variability. Ophthalmology. 1994;101(5): 866–871. 8190472 10.1016/s0161-6420(94)31246-2

[6] Chen J, Ma Z, Jiao X, Fariss R, Kantorow WL, Kantorow M, et al Mutations in FYCO1 cause autosomal-recessive congenital cataracts. Am J Hum Genet. 2011;88(6): 827–838. 10.1016/j.ajhg.2011.05.008 21636066 PMC3113247

[7] Hejtmancik JF. Congenital cataracts and their molecular genetics. Semin Cell Dev Biol. 2008;19(2): 134–149. 18035564 10.1016/j.semcdb.2007.10.003 PMC2288487

[8] Brown AL. Hereditary cataract. Am J Ophthal. 1924;7(36–38): 1924.

[9] Qi Y, Jia H, Huang S, Lin H, Gu J, Su H, et al A deletion mutation in the betaA1/A3 crystallin gene (CRYBA1/A3) is associated with autosomal dominant congenital nuclear cataract in a Chinese family. Hum Genet. 2004;114(2): 192–197. 14598164 10.1007/s00439-003-1049-7

[10] Truscott RJ. Age-related nuclear cataract-oxidation is the key. Exp Eye Res. 2005;80(5): 709–725. 15862178 10.1016/j.exer.2004.12.007

[11] Heon E, Priston M, Schorderet DF, Billingsley GD, Girard PO, Lubsen N, et al The gamma-crystallins and human cataracts: a puzzle made clearer. Am J Hum Genet. 1999;65(5): 1261–1267. 10521291 10.1086/302619 PMC1288278

[12] Messina-Baas OM, Gonzalez-Huerta LM, Cuevas-Covarrubias SA. Two affected siblings with nuclear cataract associated with a novel missense mutation in the CRYGD gene. Mol Vis. 2006;12: 995–1000. 16943771

[13] Mackay DS, Andley UP, Shiels A. Cell death triggered by a novel mutation in the alphaA-crystallin gene underlies autosomal dominant cataract linked to chromosome 21q. Eur J Hum Genet. 2003;11(10): 784–793. 14512969 10.1038/sj.ejhg.5201046

[14] Pauli S, Soker T, Klopp N, Illig T, Engel W, Graw J. Mutation analysis in a German family identified a new cataract-causing allele in the CRYBB2 gene. Mol Vis. 2007;13: 962–967. 17653036 PMC2774456

[15] Willoughby CE, Shafiq A, Ferrini W, Chan LL, Billingsley G, Priston M, et al CRYBB1 mutation associated with congenital cataract and microcornea. Mol Vis. 2005;11: 587–593. 16110300

[16] Gu Z, Ji B, Wan C, He G, Zhang J, Zhang M, et al A splice site mutation in CRYBA1/A3 causing autosomal dominant posterior polar cataract in a Chinese pedigree. Mol Vis. 2010;16: 154–160. 20142846 PMC2817011

[17] Wang KJ, Wang S, Cao NQ, Yan YB, Zhu SQ. A novel mutation in CRYBB1 associated with congenital cataract-microcornea syndrome: the p.Ser129Arg mutation destabilizes the betaB1/betaA3-crystallin heteromer but not the betaB1-crystallin homomer. Hum Mutat. 2011;32(3): E2050–2060. 10.1002/humu.21436 21972112 PMC3087119

[18] Weisschuh N, Aisenbrey S, Wissinger B, Riess A. Identification of a novel CRYBB2 missense mutation causing congenital autosomal dominant cataract. Mol Vis. 2012;18: 174–180. 22312185 PMC3272051

[19] Yao K, Jin C, Zhu N, Wang W, Wu R, Jiang J, et al A nonsense mutation in CRYGC associated with autosomal dominant congenital nuclear cataract in a Chinese family. Mol Vis. 2008;14: 1272–1276. 18618005 PMC2447816

[20] Wang B, Yu C, Xi YB, Cai HC, Wang J, Zhou S, et al A novel CRYGD mutation (p.Trp43Arg) causing autosomal dominant congenital cataract in a Chinese family. Hum Mutat. 2011;32(1): E1939–1947. 10.1002/humu.21386 21031598 PMC3035819

[21] Su D, Guo Y, Li Q, Guan L, Zhu S, Ma X. A novel mutation in CRYAA is associated with autosomal dominant suture cataracts in a Chinese family. Mol Vis. 2012;18: 3057–3063. 23288997 PMC3534140

[22] Zhang LY, Yam GH, Fan DS, Tam PO, Lam DS, Pang CP. A novel deletion variant of gammaD-crystallin responsible for congenital nuclear cataract. Mol Vis. 2007;13: 2096–2104. 18079686

[23] Graw J. Genetics of crystallins: cataract and beyond. Exp Eye Res. 2009;88(2): 173–189. 10.1016/j.exer.2008.10.011 19007775

[24] Vendra VP, Agarwal G, Chandani S, Talla V, Srinivasan N, Balasubramanian D. Structural integrity of the Greek key motif in betagamma-crystallins is vital for central eye lens transparency. PloS one. 2013;8(8): e70336 10.1371/journal.pone.0070336 23936409 PMC3735602

[25] Talla V, Srinivasan N, Balasubramanian D. Visualization of in situ intracellular aggregation of two cataract-associated human gamma-crystallin mutants: lose a tail, lose transparency. Invest Ophthalmol Vis Sci. 2008;49(8): 3483–3490. 10.1167/iovs.07-1114 18421082

[26] Basak A, Bateman O, Slingsby C, Pande A, Asherie N, Ogun O, et al High-resolution X-ray crystal structures of human gammaD crystallin (1.25 A) and the R58H mutant (1.15 A) associated with aculeiform cataract. J Mol Biol. 2003;328(5): 1137–1147. 12729747 10.1016/s0022-2836(03)00375-9

[27] Cocquerel L, Wychowski C, Minner F, Penin F, Dubuisson J. Charged residues in the transmembrane domains of hepatitis C virus glycoproteins play a major role in the processing, subcellular localization, and assembly of these envelope proteins. J Virol. 2000;74(8): 3623–3633. 10729138 10.1128/jvi.74.8.3623-3633.2000 PMC111872

