# Supplementary figures and images for: A Novel Insertion Variant of CRYGD Is Associated with Congenital Nuclear Cataract in a Chinese Family
Source: PLoS One. 2015 Jul 6;10(7):e0131471. doi: 10.1371/journal.pone.0131471 (PMC4493073; doi:10.1371/journal.pone.0131471)

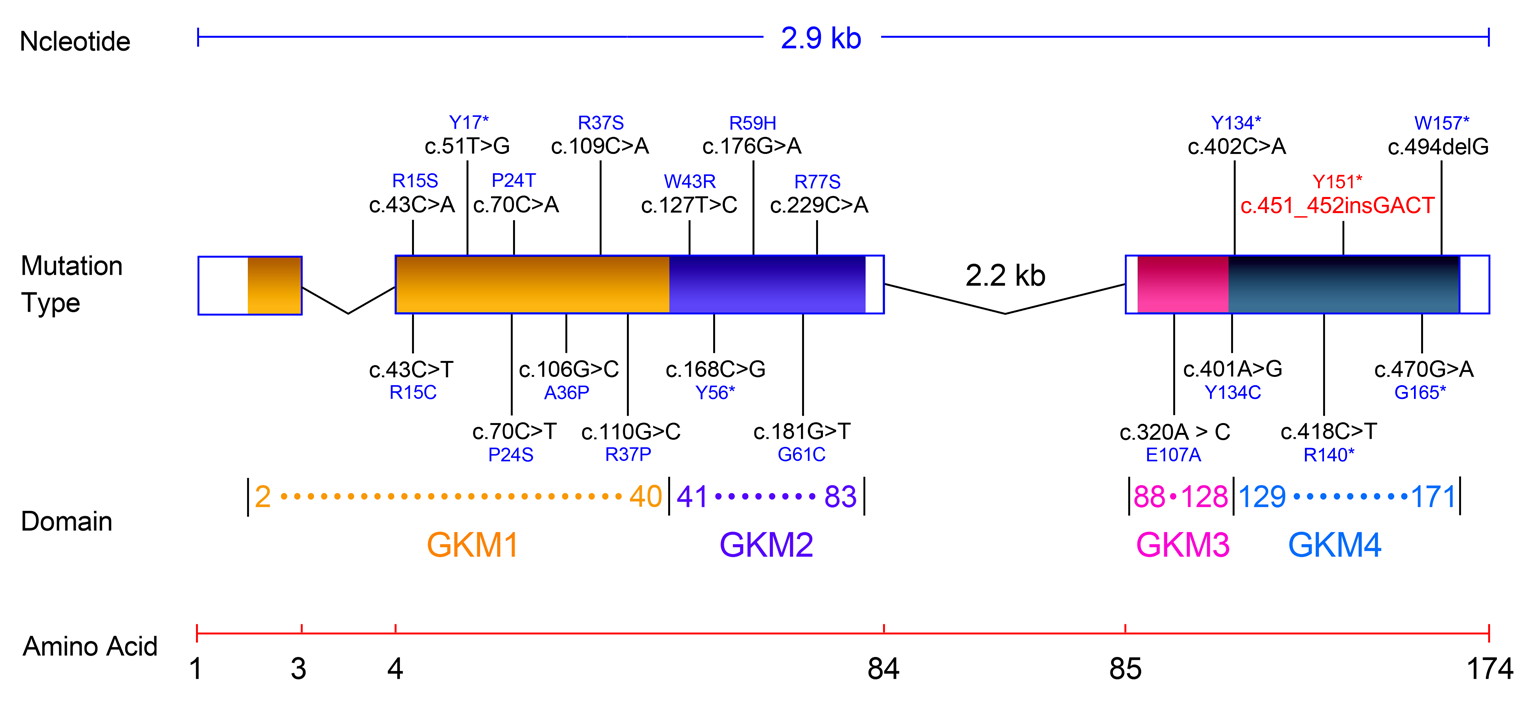

Supplement: S1 Fig — (TIF) [file pone.0131471.s001.tif]

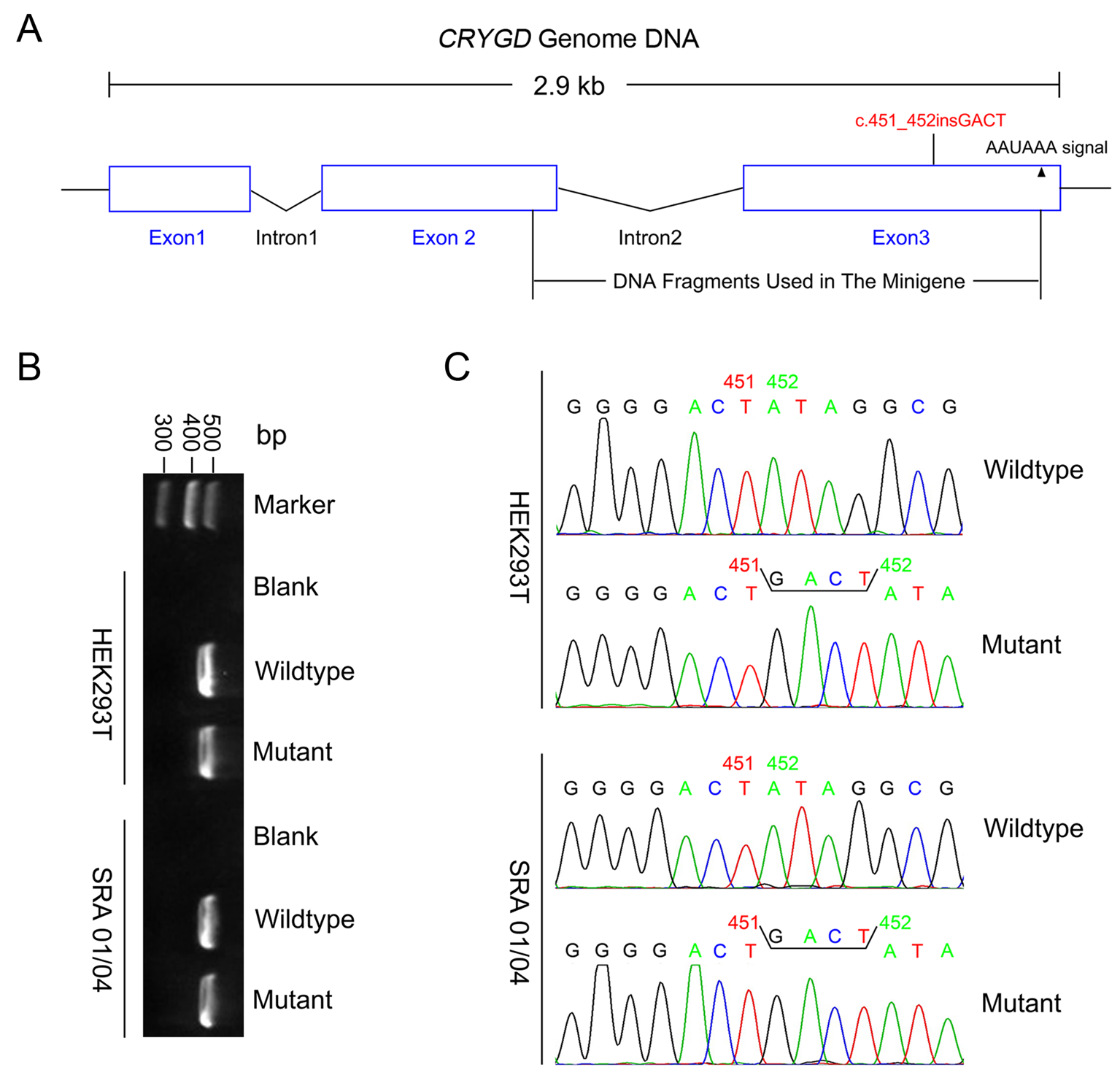

Supplement: S1 File — The schematic diagram of DNA fragments used in the constructs (Figure A). Agarose gel electrophoresis showing no difference in the PCR fragments between wildtype and mutant (Figure B). Sequence chromatogram showing the c.451_452insGACT mutation has no effect on splicing (Figure C). (TIF) [file pone.0131471.s002.tif]
