# Supplementary material for: A Novel Insertion Variant of CRYGD Is Associated with Congenital Nuclear Cataract in a Chinese Family
Source: PLoS One. 2015 Jul 6;10(7):e0131471. doi: 10.1371/journal.pone.0131471 (PMC4493073; doi:10.1371/journal.pone.0131471)
Supplement: S1 Table — (PDF) [file pone.0131471.s003.pdf]

S1 Table. Clinical information of the family members

ID	Age (years)	Sex	Type of Cataract	Age of Diagnosis (years)	BCVA Before Surgery	BCVA after Surgery	Age of Surgery (years), Lens
II5	55	F	Unaffected	—	R:0.5, L:0.6	—	No surgery
II6	55	M	Unaffected	—	R:0.8, L:0.8	—	No surgery
II7	53	F	Bilateral, Nuclear	20	R:0.1, L:0.12	R:0.2, L:0.2	36, Pseudophakia
III1	31	M	Unaffected	—	R:0.8, L:0.8	—	No surgery
III2	28	F	Bilateral, Nuclear	15	R:0.12, L:0.12	R:0.25, L:0.3	19, Pseudophakia
III3	26	M	Unaffected	—	R:0.6, L:0.8	—	No surgery
IV1	2	F	Bilateral, Nuclear	4 months	NA	NA	4 months, Pseudophakia

F, female; L, left; M, male; R, right; BCVA, best-corrected visual acuity; NA, not available.
