# Supplementary material for: A Novel Insertion Variant of CRYGD Is Associated with Congenital Nuclear Cataract in a Chinese Family
Source: PLoS One. 2015 Jul 6;10(7):e0131471. doi: 10.1371/journal.pone.0131471 (PMC4493073; doi:10.1371/journal.pone.0131471)
Supplement: S2 Table — (PDF) [file pone.0131471.s004.pdf]

S2 Table. γD-crystallin mutations, related phenotypes and functional changes

Mutation Type	Nucleotide & Amino Acid Change#	Cataract Phenotype	Protein characteristics	Reference
**Missense**	c.43C>A (p.Arg15Ser)	Coralliform	§	[1]
**Missense**	c.43C>T (p.Arg15Cys)	Punctate	§	[1, 2]
**Nonsense**	c.51T>G (p.Tyr17*)	Adult-onset, NA	NA	[3]
**Missense**	c.70C>A (p.Pro24Thr)	llamellar/Cerulean (and coralliform)/Aculeiform	§	[1, 4-6]
**Missense**	c.70C>T (p.Pro24Ser)	Irregular opacities	NA	[7]
**Missense**	c.106G>C (p.Ala36Pro)	Nuclear	NA	[8]
**Missense**	c.109C>A (p.Arg37Ser)	Nuclear/Crystal-like	NA	[9, 10]
**Missense**	c.110G>C (p.Arg37Pro)	Nuclear	NA	[11]
**Missense**	c.127T>C (p.Trp43Arg)	Nuclear	%	[12]
**Nonsense**	c.168C>G (p.Tyr56*)	Nuclear & Lamellar	NA	[13]
**Missense**	c.176G>A (p.Arg59His)	Aculeiform	NA	[14]
**Missense**	c.181G>T (p.Gly61Cys)	Coralliform	§	[1, 15]
**Missense**	c.229C>A (p.Arg77Ser)	Anterior polar coronary	NA	[16]
**Missense**	c.320A > C (p.Glu107Ala)	Nuclear	NA	[17]
**Missense**	c.401A>G (p.Tyr134Cys)	Lamellar/Pulvurulent/Nuclear	NA	[18]
**Nonsense**	c.402C>A (p.Tyr134*)	NA	NA	[19]
**Nonsense**	c.418C>T (p.Arg140*)	Nuclear (with hyperopia, strabismus)	NA	[3, 20]
**Insertion**	c.451_452insGACT (p.Tyr151*)	Nuclear	£	Present Study
**Nonsense**	c.470G>A (p.Trp157*)	Nuclear	NA	[4]
**Deletion**	c.494delG (p.Gly165*)	Nuclear	£	[1, 21]

Note: The nomenclature for the protein sequence variants of CRYGD is based on ***Recommendations for the description of protein sequence variants (v2.0)*** (http://www.hgvs.org/mutnomen/recs-prot.html), in which the Methionine encoded by the start codon is numbered as residue 1.

NA, not available; §, reduced solubility; %, unstable; £, reduced solubility and mis-localization.

References

1. Zhang LY, Gong B, Tong JP, Fan DS, Chiang SW, Lou D, et al. A novel gammaD-crystallin mutation causes mild changes in protein properties but leads to congenital coralliform cataract. Mol Vis. 2009;15: 1521-1529.
2. Stephan DA, Gillanders E, Vanderveen D, Freas-Lutz D, Wistow G, Baxevanis AD, et al. Progressive juvenile-onset punctate cataracts caused by mutation of the gammaD-crystallin gene. Proc Natl Acad Sci U S A. 1999;96(3): 1008-1012.
3. Reis LM, Tyler RC, Muheisen S, Raggio V, Salviati L, Han DP, et al. Whole exome sequencing in dominant cataract identifies a new causative factor, CRYBA2, and a variety of novel alleles in known genes. Hum Genet. 2013;132(7): 761-770.
4. Santhiya ST, Shyam Manohar M, Rawlley D, Vijayalakshmi P, Namperumalsamy P, Gopinath PM,

et al. Novel mutations in the gamma-crystallin genes cause autosomal dominant congenital cataracts. J Med Genet. 2002;39(5): 352-358.

5. Khan AO, Aldahmesh MA, Ghadhfan FE, Al-Mesfer S, Alkuraya FS. Founder heterozygous P23T CRYGD mutation associated with cerulean (and coralliform) cataract in 2 Saudi families. Mol Vis. 2009;15: 1407-1411.

6. Vanita V, Singh D. A missense mutation in CRYGD linked with autosomal dominant congenital cataract of aculeiform type. Mol Cell Biochem. 2012;368(1-2): 167-172.

7. Plotnikova OV, Kondrashov FA, Vlasov PK, Grigorenko AP, Ginter EK, Rogaev EI. Conversion and compensatory evolution of the gamma-crystallin genes and identification of a cataractogenic mutation that reverses the sequence of the human CRYGD gene to an ancestral state. Am J Hum Genet. 2007;81(1): 32-43.

8. Sun W, Xiao X, Li S, Guo X, Zhang Q. Mutation analysis of 12 genes in Chinese families with congenital cataracts. Mol Vis. 2011;17: 2197-2206.

9. Kmoch S, Brynda J, Asfaw B, Bezouska K, Novak P, Rezacova P, et al. Link between a novel human gammaD-crystallin allele and a unique cataract phenotype explained by protein crystallography. Hum Mol Genet. 2000;9(12): 1779-1786.

10. VanderVeen DK, Andrews C, Nihalani BR, Engle EC. Crystalline cataract caused by a heterozygous missense mutation in gammaD-crystallin (CRYGD). Mol Vis. 2011;17: 3333-3338.

11. Wang L, Chen X, Lu Y, Wu J, Yang B, Sun X. A novel mutation in gammaD-crystallin associated with autosomal dominant congenital cataract in a Chinese family. Mol Vis. 2011;17: 804-809.

12. Wang B, Yu C, Xi YB, Cai HC, Wang J, Zhou S, et al. A novel CRYGD mutation (p.Trp43Arg) causing autosomal dominant congenital cataract in a Chinese family. Hum Mutat. 2011;32(1): E1939-1947.

13. Santana A, Waiswol M, Arcieri ES, Cabral de Vasconcellos JP, Barbosa de Melo M. Mutation analysis of CRYAA, CRYGC, and CRYGD associated with autosomal dominant congenital cataract in Brazilian families. Mol Vis. 2009;15: 793-800.

14. Heon E, Priston M, Schorderet DF, Billingsley GD, Girard PO, Lubsen N, et al. The gamma-crystallins and human cataracts: a puzzle made clearer. Am J Hum Genet. 1999;65(5): 1261-1267.

15. Li F, Wang S, Gao C, Liu S, Zhao B, Zhang M, et al. Mutation G61C in the CRYGD gene causing autosomal dominant congenital coralliform cataracts. Mol Vis. 2008;14: 378-386.

16. Roshan M, Vijaya PH, Lavanya GR, Shama PK, Santhiya ST, Graw J, et al. A novel human CRYGD mutation in a juvenile autosomal dominant cataract. Mol Vis. 2010;16: 887-896.

17. Messina-Baas OM, Gonzalez-Huerta LM, Cuevas-Covarrubias SA. Two affected siblings with nuclear cataract associated with a novel missense mutation in the CRYGD gene. Mol Vis. 2006;12: 995-1000.

18. de Figueiredo ES, Giordano GG, Tavares A, da Silva MJ, de Vasconcellos JP, Arieta CE, et al. Novel human CRYGD rare variant in a Brazilian family with congenital cataract. Mol Vis. 2011;17: 2207-2211.

19. Hansen L, Yao W, Eiberg H, Kjaer KW, Baggesen K, Hejtmancik JF, et al. Genetic heterogeneity in microcornea-cataract: five novel mutations in CRYAA, CRYGD, and GJA8. Invest Ophthalmol Vis Sci. 2007;48(9): 3937-3944.

20. Devi RR, Yao W, Vijayalakshmi P, Sergeev YV, Sundaresan P, Hejtmancik JF. Crystallin gene mutations in Indian families with inherited pediatric cataract. Mol Vis. 2008;14: 1157-1170.

21. Zhang LY, Yam GH, Fan DS, Tam PO, Lam DS, Pang CP. A novel deletion variant of gammaD-

crystallin responsible for congenital nuclear cataract. Mol Vis. 2007;13: 2096-2104.
